# Determination of time lag by accurate monitoring of pressure decay in a new generation constant volume system

**DOI:** 10.1016/j.mex.2024.102858

**Published:** 2024-07-10

**Authors:** Peter Leszczynski, Zheng Cao, Haoyu Wu, Jules Thibault, Boguslaw Kruczek

**Affiliations:** Department of Chemical and Biological Engineering, University of Ottawa, 161 Louis Pasteur St., Ottawa, Canada K1N 6N5

**Keywords:** Membrane characterization, Constant volume systems, Time-lag method, Pressure decay, Differential pressure transducer, Determination of membrane time lag based on pressure decay in a constant volume system

## Abstract

The time-lag method is the standard approach for evaluating membrane permeability, diffusivity and solubility in a single gas permeation experiment. The conventional time-lag method relies on accurately monitoring the pressure rise in a constant volume downstream from the membrane following a change in pressure upstream from the membrane. The same information could be extracted from the upstream pressure decay in the same time-lag experiment. However, accurately monitoring the pressure decay presents a challenge due to the resolution limitations of absolute pressure transducers. If the membrane was characterized based on pressure decay, a mass spectrometer could be used to simultaneously monitor the composition of the gas permeating from the membrane, opening the time-lag method to gas mixtures. Also, the simultaneous monitoring of pressure rise and decay could provide additional information about gas transport in the membrane, which is critical for more complex membrane materials.

• The resolution challenge was overcome by splitting the upstream volume into the working and reference volumes and monitoring pressure decay using a differential pressure transducer between the two volumes.
• The validity of the measured pressure decay was confirmed by the unique relation between the upstream and downstream time lags for a commercial PDMS membrane.

## Graphical abstract

**Figure:**
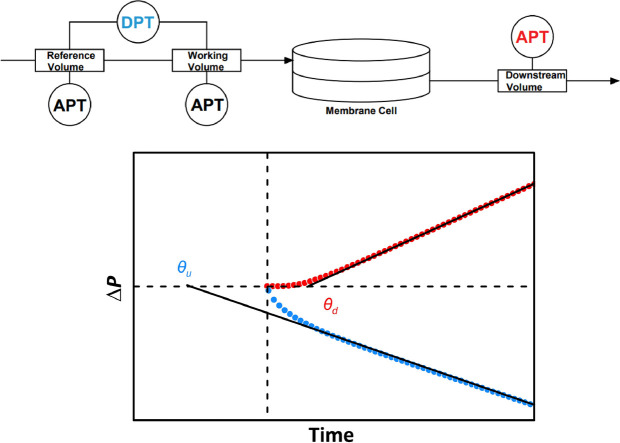

Specifications table

**Table:** 

Subject area:	Chemical Engineering
More specific subject area:	Gas transport properties in polymer membranes
Name of your method:	Determination of membrane time lag based on pressure decay in a constant volume system.
Name and reference of original method:	M. Al-Ismaily, J.G. Wijmans, B. Kruczek, A shortcut method for faster determination of permeability coefficient from time lag experiments, J. Memb. Sci. 423–424 (2012). https://doi.org/10.1016/j.memsci.2012.08.009
Resource availability:	Custom-made constant volume (CV) system, major components include: • One capacitance-based differential pressure transducer (CCR Process Products, MKS698A) • Two absolute pressure transducers 0 to 10 Torr (CCR Process Products MKS627F11TBC1B) • Two absolute pressure transducers 0–2000 Torr (CCR Process Products, MKS627F13TBC1B) • Rotary vacuum pump (Edwards RV3, A65201906) • Turbomolecular pump (Edwards TS85D1002) • Remotely operated valves: valve body + actuator (Swagelok SS-4BK-VCR-1D & SS-4BK-1C-K10)

## Background

Permeability (*P*), diffusivity (*D*), and solubility (*S*) coefficients are the critical properties of membrane materials. They are determined in a single time-lag experiment performed in a constant volume (CV) system. A degassed membrane from the tested material is exposed to a step pressure increase while the resulting pressure rise at the permeate side of the membrane is monitored. The tangent of the linear portion of the pressure rise is directly proportional to *P.* The extrapolation of that linear portion to the time axis yields a time lag (*θ_d_*), which is related to the diffusivity:

(1) $$\theta_{d}=\frac{L^{2}}{6D}$$

where *L* is the membrane thickness. According to the solution-diffusion model, the ratio of *P* and *D* represents *S*.

Following the change in feed pressure, the time-lag method could also rely on monitoring the pressure decay in a constant volume upstream from the membrane. The corresponding expression for the upstream time lag (*θ_u_*) is given by [1]:

(2) $$\theta_{u}=-\frac{L^{2}}{3D}$$

The measurements in the CV system require accurate monitoring of the pressure change with time. Typically, the resolution of a high-quality absolute pressure transducer is 1/10,000 of its maximum reading. When monitoring the pressure rise at a high vacuum, it is sufficient to use the pressure transducer with a maximum reading of 1 Torr with the corresponding resolution of 0.0001 Torr. On the other hand, a pressure transducer with a maximum reading of at least 1000 Torr would be required to monitor the pressure decay in a time-lag experiment. The corresponding resolution of 0.1 Torr is insufficient when characterizing low-permeability materials.

Al-Ismaily et al. [2] addressed the resolution problem of absolute pressure transducers by adopting the concept of a two-tank volume developed by Arkilic et al. [3]. In a two-tank volume system, one tank is a reference volume, and the other is a working volume. The microflow in or out of the working volume is measured by comparing the resulting pressure change relative to the constant reference volume pressure using a differential pressure transducer (DPT). For microflows, the maximum range of DPT can be small (e.g. 1 Torr), allowing for very high resolutions. Because a step change in feed pressure is needed to initiate time-lag experiments, the working volume must be split to maintain the membrane at a high vacuum before the experiment. This creates a dead volume that includes the upstream part of the membrane cell. Upon initiation of the experiment, the gas expands into the dead volume, resulting in a pressure drop greater than the range of the DPT, prohibiting data acquisition. As a result, Al-Ismaily et al. started experiments when the reference and working volumes were connected, and the progress of the experiments could only be monitored after separating the two volumes. In addition, closing the valve between the two volumes was associated with a “compression effect” and led to $|\theta_{u}/\theta_{d}|$ much greater than the theoretical value of 2. Moreover, the feed pressure in time-lag experiments was limited to 175 Torr [2]. To overcome these challenges, we designed the next-generation CV system.

Simultaneous monitoring of pressure decay and rise opens enormous possibilities for characterizing complex membrane materials with strongly nonlinear sorption behaviour. For example, it can completely characterize dual-mode sorption membranes based on instantaneous upstream and downstream time lags and their ratio without additional sorption experiments [4]. To demonstrate the capability of monitoring genuine pressure decay in the new CV system requires selecting the membrane material for which $|\theta_{u}/\theta_{d}|\;$has a unique value, and polydimethylsiloxane (PDMS), a rubbery polymer, is a material for which this ratio should equal 2 [4].

## Method details

### Equipment and materials

**Fig. 1:**
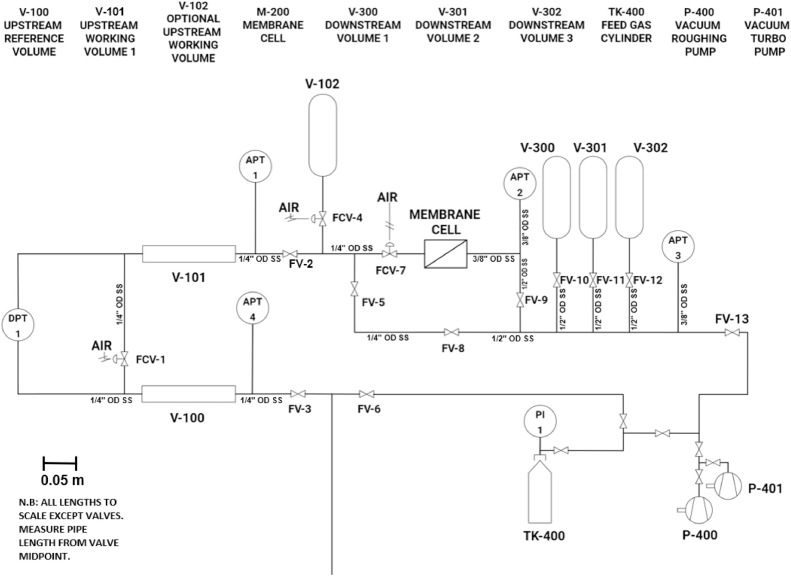
A scaled schematic of the constant volume (CV) system.

Fig. 1 presents a scaled schematic of the CV system, which comprises three distinct sections: (1) the upstream section, which is bounded by valves FV-3, FV-5, and FCV-7; (2) the membrane cell; and (3) the downstream receiver, which is bounded by the membrane cell, FV-8, and FV-13. The upstream portion of the CV system includes two sections: the working volume (V-101, *V_w_* = 89 cm^3^) and the reference volume (V-100, *V_r_* = 96 cm^3^). The valve FCV-1 separates volumes V-100 and V-101. During experiments, when FCV-7 is open, the working volume includes a dead volume (*V_ud_*) that is enclosed between FCV-7 and the upstream portion of the membrane cell. The dead volume is 11 cm^3^ but can be reduced to 4 cm^3^ by inserting a metal disk into the upstream portion of the membrane cell. This reduction in *V_ud_* helps decrease gas expansion when the experiment begins. The downstream section featured three additional volumes (V-300, V-301, and V-302) that can easily be added or removed from the downstream receiver depending on the anticipated permeability of the tested membrane [5]. When testing barrier materials, FV-9 can be closed to minimize the volume of the downstream receiver.

The membranes are characterized by monitoring the pressure rise and decay responses to a step change in feed pressure. The pressure rise is measured with 10-Torr absolute pressure transducers APT2 and APT3 (CCR Process Products Kanata, Ontario, Canada, MKS627F11TBC1B, 10^–5^ full-scale resolution, accuracy: 0.12 % of reading, range: 0 to 10 Torr). The pressure decay is measured using a pressure differential between V-100 and V-101 with a capacitance-based differential pressure transducer DPT1 (CCR Process Products Kanata, Ontario, Canada, MKS698A ±1 Torr, 10^–6^ full-scale resolution, accuracy: 0.05 % of reading). There are also two identical absolute pressure transducers, APT1 and APT4 (CCR Process Products Kanata, Ontario, Canada, MKS627F13TBC1B, 10^–5^ full-scale resolution, accuracy: 0.12 % of reading, range: 0 to 2000 Torr) to monitor the pressures in the working and reference volumes in the upstream section of the system, respectively. In principle, APT1 and APT4 (when FCV-1 is open) or APT1 (when FCV-1 is closed) can also be used to monitor the pressure decay in the upstream portion of the CV system during time-lag experiments, albeit at a much lower resolution than DPT1.

**Fig. 2:**
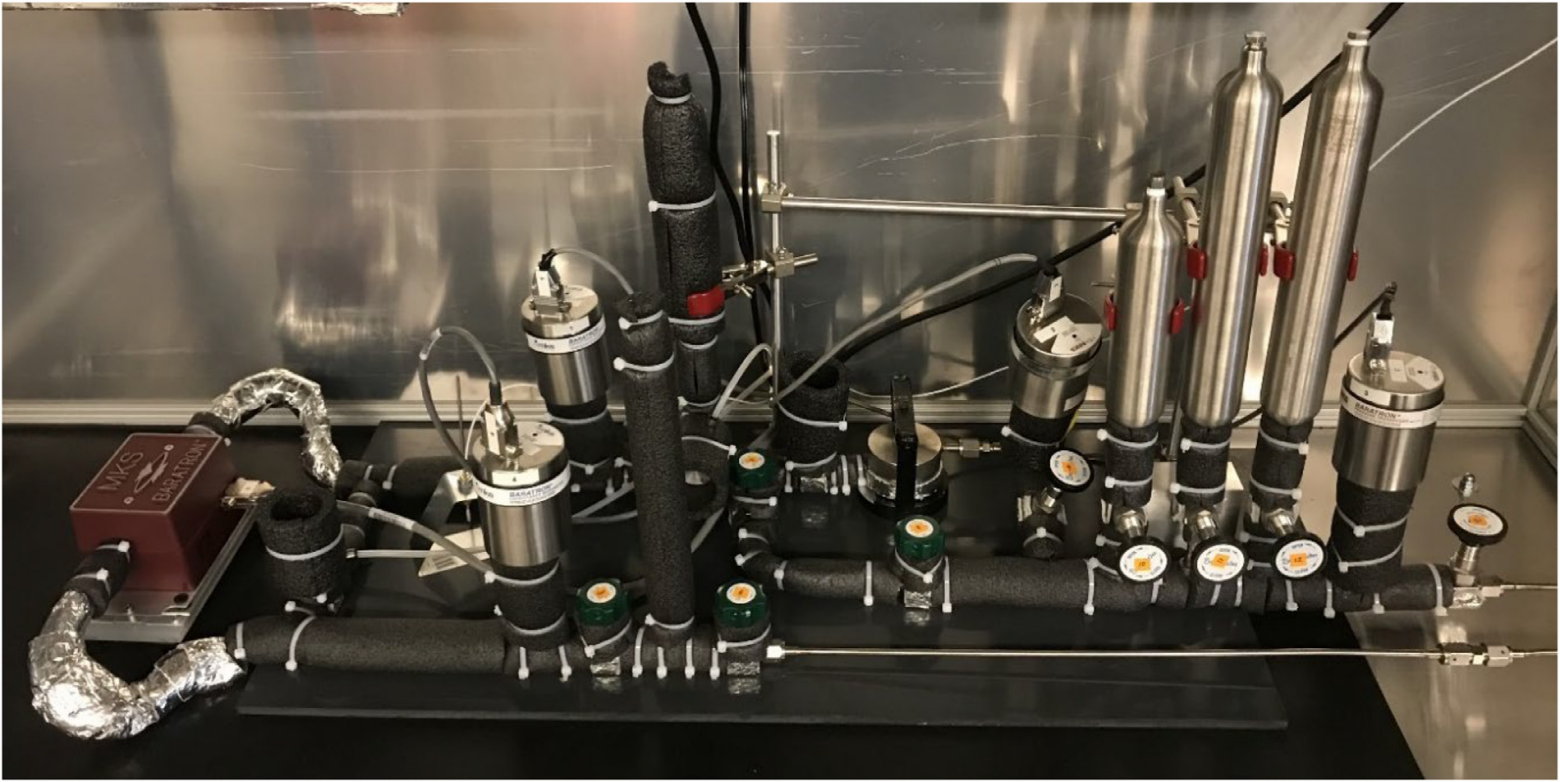
The picture of the inside of the controlled temperature chamber. Referring to Fig. 1, the components on the picture's left and right are DPT1 and APT3, respectively.

All valves in Fig. 1 were procured from Weston Valve & Fitting LTD, Mississauga, ON (Canada). The FV valves are manually operated stainless steel bellows sealed valves (Swagelok SC-4BK-VCR). The remotely-operated FCV valves comprise a valve body and an actuator (Swagelok SS-4BK-VCR-1D & SS-4BK-1C-K10). The valve connections are either brazed or sealed tight with metal gasket face seals to reduce possible sources of leaks. All components of the system, except for a rotary vacuum pump, P-400 (CANVACTECH, Ottawa, Ontario, Canada, Edwards RV3, A65201906, ultimate pressure: 1.5 × 10^–3^ Torr), a turbomolecular pump P-401 (Edwards Vacuum LLC Sanborn, New York, USA, Edwards TS85D1002, ultimate pressure: < 3.75 × 10^–10^ Torr) and a gas cylinder TK-400, are enclosed in a temperature controlled chamber. The operational temperature range of the chamber is between 273 K and 313 K. The chamber could be heated to higher temperatures, but the APTs and DPT would lose accuracy at temperatures greater than 313 K. The details of the heating and cooling components of the system are described elsewhere [5]. The picture of the system components inside the chamber is shown in Fig. 2.

The operation of the system was demonstrated using commercially available polydimethylsiloxane (PDMS) membranes (SSPM823–010–12”-SSP-M823-Platinum Cured Silicone), purchased from Interstate Specialty Products (Sutton, MA). These membranes were supplied with a white virgin Teflon backing. As per the supplier's information, the thickness of the PDMS membranes was 0.010” ± 0.002” (0.254 mm ± 0.051 mm) thickness. All experiments were performed using a 99.99 % pure N_2_ from the compressed gas cylinder, TK-400.

### Experimental method

Once the membrane was installed within the cell, the system underwent extensive evacuation to degas the membrane using two vacuum pumps, P-400 and P-401. The available valves allowed for the compartmentalization of the system. For example, when FV-13 was open, and both FV-8 and FV-9 were closed, the evacuation was confined to the downstream section of the system. The downstream part of the membrane cell was included in the evacuation process by opening FV-9. The entire membrane cell could be evacuated by opening the bypass line (with FV-8, FV5, and FCV 7 open) while keeping FV-2 and FCV-4 closed. The upstream part of the system, which includes the working and reference volumes, could be evacuated via the bypass line and by opening FV-2 (while keeping FV-3 closed). However, the working and reference volumes were evacuated directly via open FV-6 while keeping FV-13, FV-5, and FCV-7 closed. Before each gas permeation test, the leak rates in different parts of the system were evaluated by monitoring the pressure rise using the appropriate absolute pressure transducers after the system had been fully evacuated (more than 24 h). Table 1 presents typical leak rates in different parts of the system.

**Table 1:** Typical leak rates within the CV system.

Section	Bounds	Volume (cm^3^)	Leak rate (Torr/s)
Reference volume	FCV-1, FV-6	96	6.0 × 10^–7^
Total working volume	FCV-1, FCV-4, FV-5, membrane	93 (100*)	9.0 × 10^–7^
Downstream volume (tubing)	Membrane, FV-8, FV-10, FV-11, FV-12, FV-13	81	8.1 × 10^–7^

*without a metal disk in the upstream part of the membrane cell.

The leak rates in Table 1 are negligible as they were 4 to 5 orders of magnitude lower than the measured pressure decay and rise typically encountered within the upstream and downstream sections of the CV system during membrane characterization trials.

Following the completion of leak tests in different sections, the system underwent another round of evacuation. Then, the valves were closed in the following sequence: FCV-7, FV-5, FV-8 and FV-13. For the pressure rise, the downstream receiver was configured to include only the tubing. This was achieved by closing FV-10, FV-11, and FV-12 while opening FV-9. The gas from TK-400 was then introduced into V-101 and V-100 at a pressure exceeding the level desired for the time-lag experiment. FV-6 was closed, and FV-2 opened, allowing the gas to flow through FV-5 and FCV-7. FCV-1 was closed to isolate the working and reference volumes, and the gas was allowed to equilibrate thermally with the air in the chamber.

Due to a dead volume, initiating the experiment by opening FCV-7 was associated with a gas expansion much larger than 1 Torr, the upper limit of DPT-1. Therefore, before initiating the experiment, the pressure within V-100 had to be reduced by an amount equivalent to the expected pressure drop in V-101. This drop was directly proportional to the feed pressure. The relationship between the feed pressure and the expansion in V-101 was evaluated in parallel experiments using an impermeable film in the membrane cell. These experiments utilized the pressures recorded by APT-1 and APT-4. The pressure reduction within V-100 was achieved using P-400, and the rate of evacuation was regulated by partially opening valve FV-6. Once the desired pressure in V-100 was reached, FV-6 was closed, and P-400 was disengaged to minimize vibrations in the system. The time-lag experiment was then initiated by opening FCV-7, with the pressure rise and decay being simultaneously monitored and recorded in real time.

The experimental protocol described above is referred to as Method 1. This method was not feasible with the first generation of the CV system, as described in Ref. [2]. For comparison, the membranes were also tested using the previous protocol, referred to as Method 2. In Method 2, the working and reference volumes remained interconnected even after establishing the desired feed pressure in V-100 and V-101. Similar to Method 1, the experiment was initiated by opening FCV-7. However, because FCV-1 was open, DPT-1 was unable to measure the pressure decay resulting from gas permeation into the membrane. This measurement was only possible after closing FCV-1. Both FCV-1 and FCV-7 are pressure-actuated valves that can be remotely controlled, eliminating the need for manual operations within the temperature-controlled chamber during the initiation of the experiment in both methods. Moreover, in Method 2, the time interval between the opening of FCV-7 and the closing of FCV-1 could be precisely controlled, improving the repeatability of the experiments. This feature was not available in the first generation of the system [2].

### Processing of pressure decay data

**Fig. 3:**
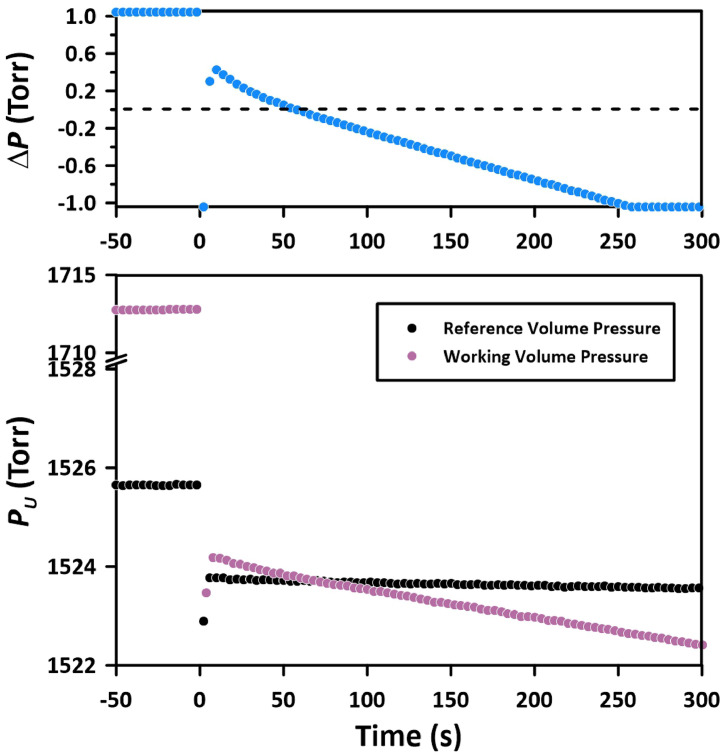
Progress of the experiment with N_2_ at 1524.2 Torr and 303 K performed using Method 1 with the dead volume of 11 cm^3^. The top panel shows the recorded pressure decay by DPT-1; the bottom part shows the corresponding absolute pressures by APT-1 in the working volume and APT-4 in the reference volume.

Fig. 3 presents the pressure decay observed in an experiment performed with N_2_ at a pressure of 1524.2 Torr and a temperature of 303 K using Method 1. The top panel of Fig. 3 shows the pressure decay recorded by DPT-1, while the bottom panel displays the corresponding absolute pressures measured by APT-1 in the working volume and APT-4 in the reference volume. The pressures were recorded for 50 s before the initiation of the experiment, as indicated by the constant pressures in the working and reference volumes when time *t* is less than 0. Before the experiment, the pressure in the working volume was 1712.8 Torr, which was 187.2 Torr higher than the pressure in the reference volume. This pressure difference was in line with the anticipated pressure drop in the working volume upon the initiation of the experiment, i.e., the pressurization of the membrane that was initially in a vacuum when *V_ud_* was 11 cm^3^.

It is observed that a of 187.2 Torr in the working volume corresponds to a 1.8 Torr pressure drop in the reference volume. The differential pressure transducer operates by measuring the change in capacitance resulting from the deflection of a metal diaphragm. This diaphragm serves to compare pressures within the working and reference volumes. Because of the large diameter of this diaphragm, even a minor displacement can lead to a non-negligible change in both volumes. As the gas expands into the dead volume and the pressure within the working volume decreases, the diaphragm shifts towards the working volume, reducing its volume, thereby increasing the reference volume. The volume change (*V_c_*) was estimated from Eq. (3).

(3) $$p_{1}V_{r}=p_{2}\left(V_{r}+V_{c}\right)$$

where *p*_1_ and *p*_2_, according to Fig. 3, were 1525.6 Torr and 1523.8 Torr, respectively, and *V_r_* = 96 cm^3^. Accordingly, *V_c_* = 0.11 cm^3^. The CCR Process Products in Kanata, Ontario, the supplier of the differential pressure transducer, confirmed this phenomenon associated with the operation of DPT-1. The volume displacements due to the sudden pressure changes can be in the order of 0.5 cm^3^. The calculated *V_c_* of 0.11 cm^3^ was less than 0.5 cm^3^. The diaphragm displacement resulting from the step change in pressure to initiate the gas permeation experiments was considered when adjusting the pressure in the reference volume using Method 1.

Immediately after initiating the gas permeation experiment, the membrane is expected to act as a semi-infinite solid. It has been shown that during this period, the pressure decay (denoted as Δ*p_u_*) should be a linear function of the square root of time [2]:

(4) $$\Delta p_{u}=-\frac{2p_{u}SRTA\sqrt{D}}{V_{u}\sqrt{\pi }}\sqrt{t}$$

where *p_u_* is the upstream (feed) pressure, *V_u_* is the total working (upstream) volume, *A* is the membrane area, *T* is the absolute temperature, and *R* is the universal gas constant. Eq. (4) implies that Δ*p_u_* << *p_u_*. The slope of Δ*p_u_* vs $\sqrt{t}$ is directly proportional to $S\sqrt{D}$, but the intercept derived from Eq. (4) is zero. Consequently, the plot Δ*p_u_* versus $\sqrt{t}$, at early times, should lead to a straight line with a nil intercept. This observation forms the foundation for correcting the pressure decay measured by DPT-1.

**Fig. 4:**
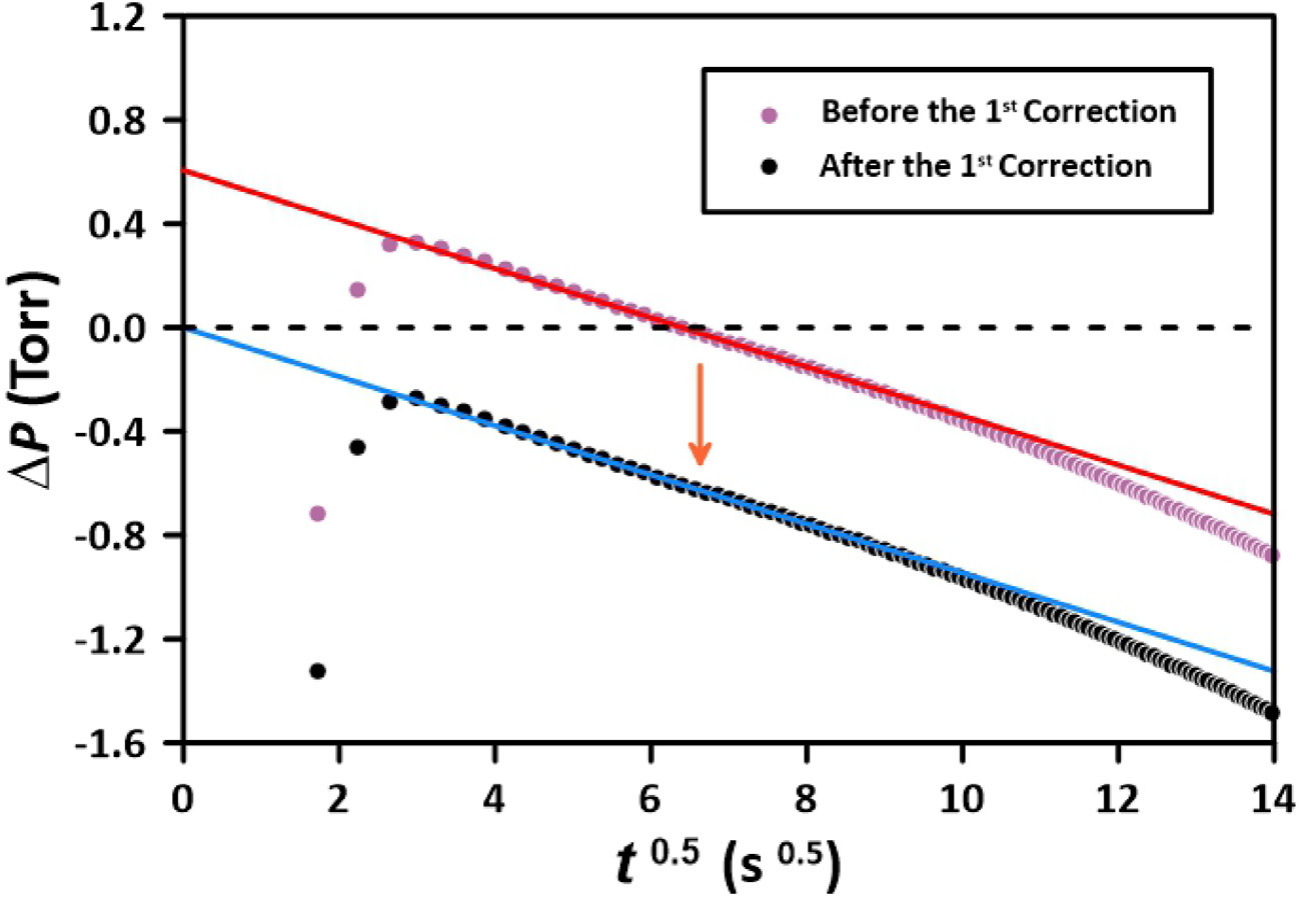
Application of the 1st correction to pressure decay data based on the expected zero intercept in the square root of the time domain. The experiment with N_2_ at 1524.2 Torr and 303 K was performed using Method 1.

Fig. 4 presents the pressure decay results, as shown in the top panel of Fig. 3 but transformed into the square root of the time domain. Except for the first few readings, the pressure decay shows the expected linear relationship. At the $\sqrt{t}$ ∼ 9, i.e., at *t* > 80 s, the pressure decay rate increases, deviating from the linear trend. This deviation indicates that the gas molecules are exiting the membrane at the permeate side, indicating that the membrane can no longer be treated as a semi-infinite solid. The original pressure decay data leads to an intercept of approximately 0.6 Torr. This intercept results from extrapolating the linear portion of the pressure decay versus the square root of time. Therefore, all pressure decay data was corrected by this factor so that the intercept would be zero. We will refer to this data manipulation as the 1st correction. The first three points in Fig. 4 fall below the expected straight line, and they correspond to the points which do not follow the general trend in the top panel of Fig. 3. Although Method 1 allows recording the pressure decay right after the initiation of the experiment, the pressure decay becomes the linear function of time only after 5 to 6 s ($\sqrt{t}$ ∼ 2.4). The *Limitations* section will further discuss why the early pressure decay data is unreliable.

**Fig. 5:**
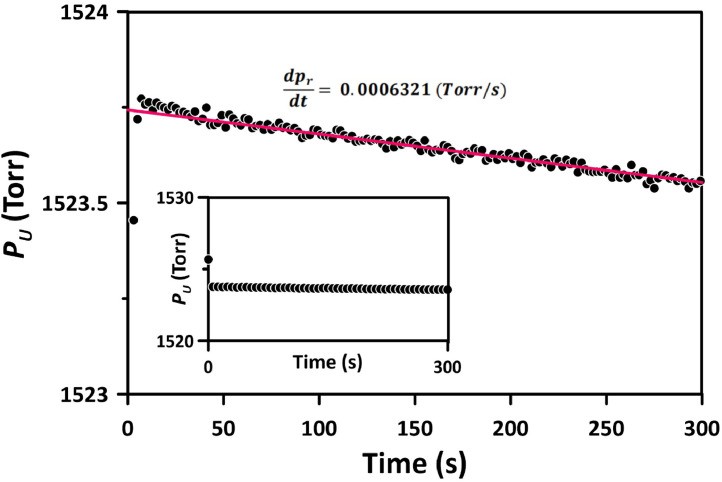
Pressure in the reference volume as a function of time in the experiment with N_2_ at 1524.2 Torr and 303 K using Method 1. The inset shows the exact relation between the reference pressure and time in the scale used in Fig. 3.

The displacement of the diaphragm at the initiation of the experiment, which was responsible for a 1.8 Torr pressure drop in the reference volume, was not exclusive to scenarios involving a sudden change in pressure. A micro-displacement of the diaphragm inside the differential pressure transducer towards the working volume occurred continuously as long as the pressure in the working volume decreased because of gas permeation into the membrane. Fig. 5 presents a magnified view of the lower panel of Fig. 3. The inset in Fig. 5 reproduces the original *p_r_* versus *t* from Fig. 3. When the pressure scale is reduced, it becomes apparent that *p_r_* decreases with *t*. Despite the low resolution (*p_r_* was monitored using APT-4), it is evident that this decrease generally follows a linear function of time. The linear regression of the pressure data in Fig. 5 gives $dp_{r}/dt$ = 0.0006321 Torr/s. Knowing $dp_{r}/dt$, the corresponding pressure increase in the working volume due to the diaphragm displacement ($dp_{wd}/dt$) can be approximated by:

(5) $$\frac{dp_{wd}}{dt}=\left|\frac{dp{}_{r}}{dt}\right|\frac{V_{r}}{V_{w}}$$

Assuming that for a given feed pressure, the pressure changes, $dp_{r}/dt$ and $dp_{wd}/dt$, were constant, the actual upstream pressure can be calculated using:

(6) $$p_{u}=p_{m}+\left(\left|\frac{dp{}_{r}}{dt}\right|+\frac{dp_{wd}}{dt}\right)t$$

where $p_{m}$ is the measured pressure in the working volume. As a result of the 2nd correction, the pressure decay rate increases.

**Fig. 6:**
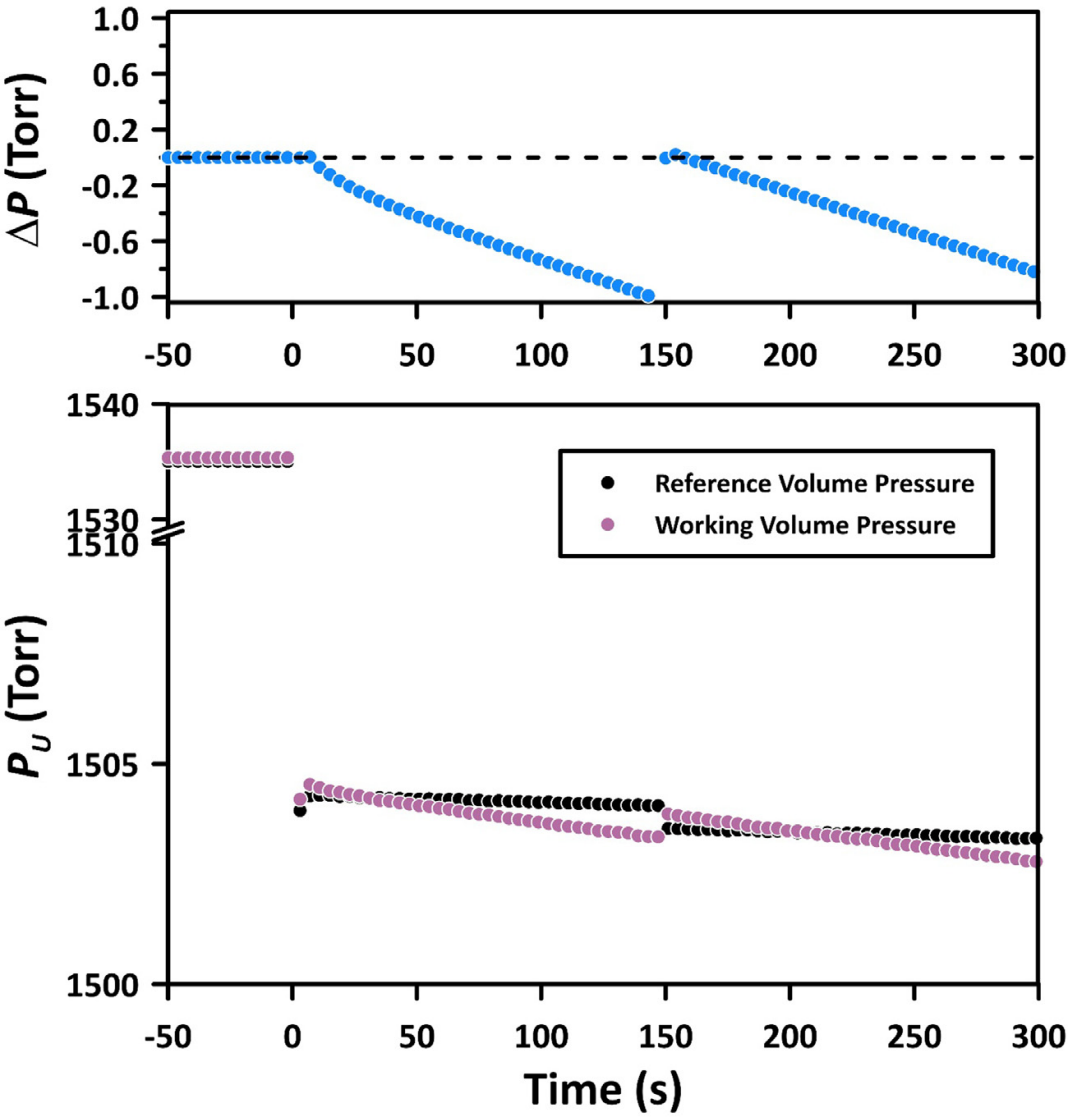
Progress of the experiment with N_2_ at 1504 Torr and 303 K performed using Method 2 with the dead volume of 4 cm^3^. The top panel shows the recorded pressure decay by DPT-1; the bottom panel shows the corresponding absolute pressures by APT-1 in the working volume and APT-4 in the reference volume.

**Fig. 7:**
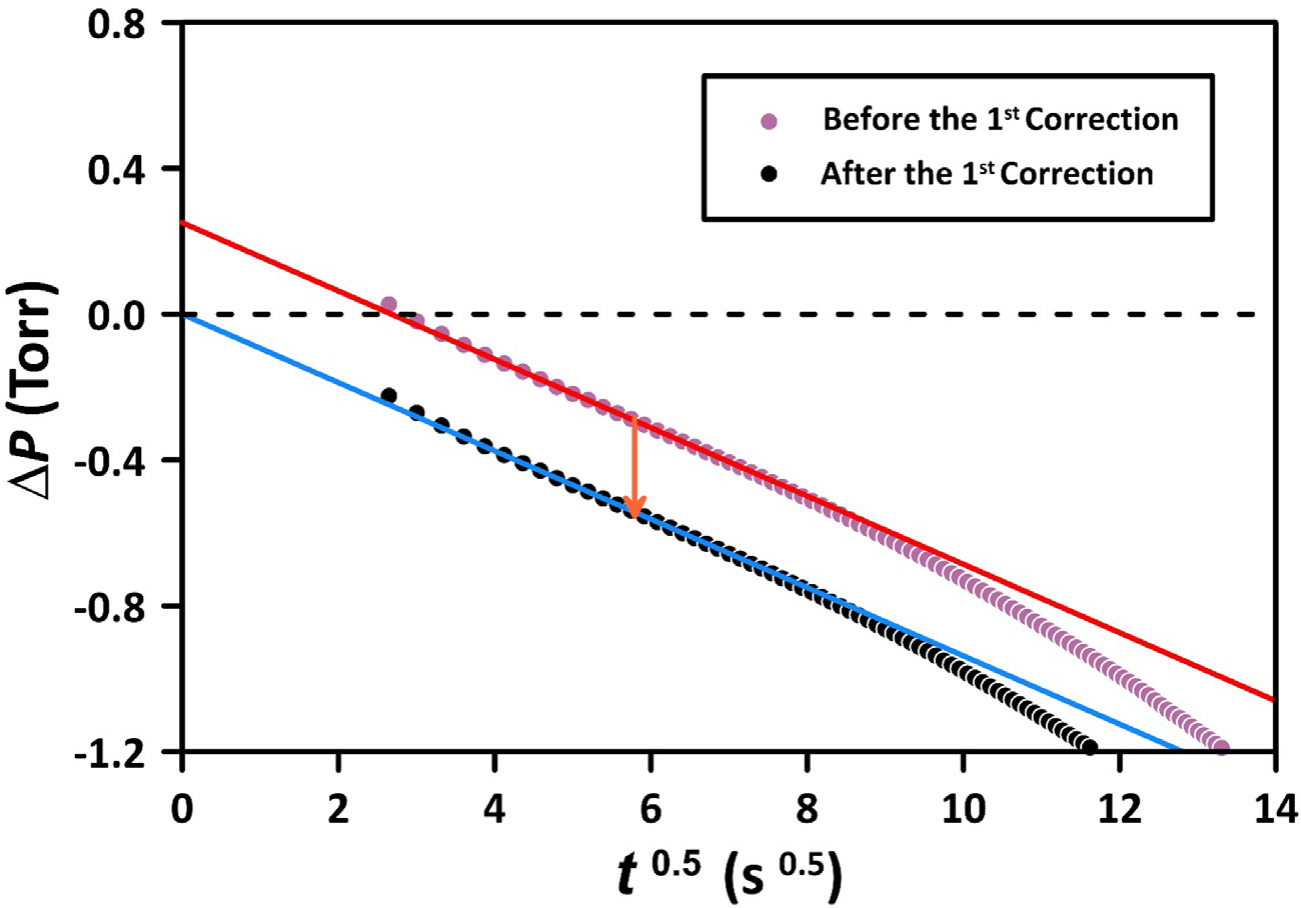
Application of the 1st correction to pressure decay data based on the expected zero intercept in the square root of the time domain. The experiment with N_2_ at 1504 Torr and 303 K was performed using Method 2.

Fig. 6, akin to Fig. 3, presents the pressure decay in an experiment performed using Method 2 with N_2_ at a pressure of 1504 Torr and a temperature of 303 K. FCV-1 was opened 5 s after initiating the experiment (i.e., upon opening FCV-7). The top panel of Fig. 6 shows the pressure decay recorded by DPT-1, while the bottom panel presents the corresponding absolute pressures measured by APT-1 in V-101 and APT-4 in V-100. Before starting the experiment, the pressure was 1535 Torr. The significantly smaller pressure drop observed at the start of the experiment in Fig. 6 (21 Torr) compared to that in Fig. 3 (187.2 Torr) can be attributed to two factors. Firstly, V-100 and V-101 were connected, resulting in a combined volume of 185 cm^3^, compared to 89 cm^3^ when V-100 and V-101 were separated in Method 1. Secondly, the dead volume in Fig. 6 was 4 cm^3^, which is smaller than the 11 cm^3^ in Fig. 3. Despite the considerably smaller pressure drop, the original pressure decay data still required the application of the 1st correction. The application of the 1st correction is shown in Fig. 7.

When compared to Fig. 4, the intercept in Fig. 6, which represents the 1st correction, is significantly smaller, being 0.14 Torr as opposed to 0.6 Torr in Fig. 6. Also, after closing FCV-1, the deviation from the semi-infinite model is much less pronounced than in Method 1. Interestingly, the initial deviation from the semi-infinite is slightly positive, unlike the negative deviation in Fig. 4. Similarly to Method 1 (Fig. 5), after closing FCV-1, the pressure in the reference volume decreased linearly with time due to the gas permeation into the membrane. Therefore, the 2nd correction of the pressure decay data, as described by (5), (6) is also required in Method 2.

## Method validation

**Fig. 8:**
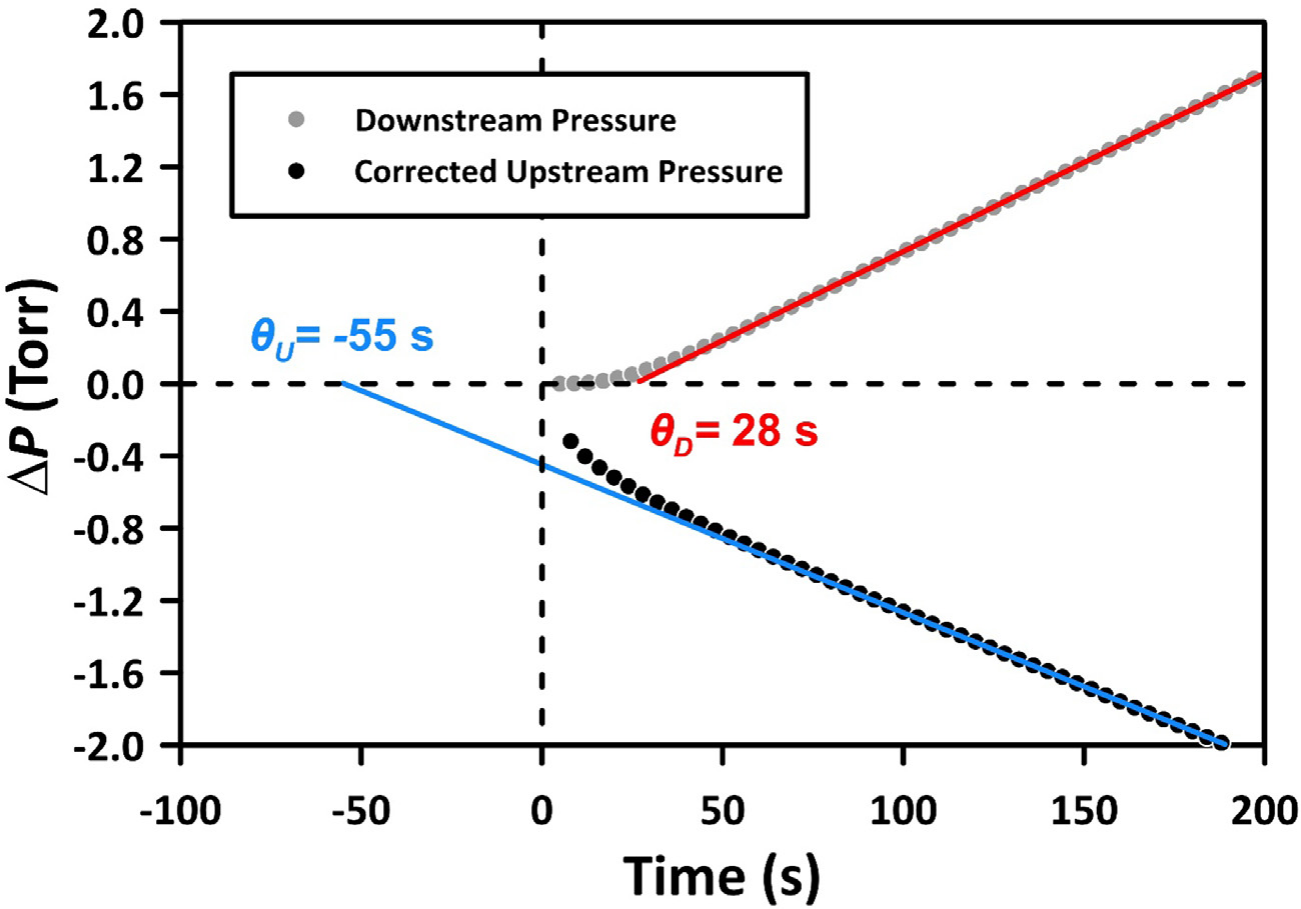
Application of the 1st correction to pressure decay data based on the expected zero intercept in the square root of the time domain. The experiment with N_2_ at 1504 Torr and 303 K was performed using Method 2.

In addition to the pressure decay results, our CV system allows for the simultaneous recording of conventional pressure rise data in the downstream part of the system. Unlike the pressure decay, the pressure rise data requires no corrections. Since PDMS is a rubbery polymer, there is a unique relationship between the downstream (as per Eq. (1)) and the upstream (as per Eq. (2)) time lags. More specifically, $|\theta_{u}/\theta_{d}|=2.$ Fig. 8 presents the progress of the experiment conducted with N_2_ at 1504 Torr and 303 K. This figure includes the corrected pressure decay data collected using Method 2, as described in the previous section, along with the “as recorded” pressure rise data. The tangent of the linear portion of the pressure decay and pressure rise data, when extended to the time axis, determines $\theta_{u}$ and $\theta_{d}$, which are −55 s and 28 s, respectively. Therefore, $|\theta_{u}/\theta_{d}|=$ 1.96, which is close to the expected value of 2. It is important to note the near equivalence of $|\theta_{u}/\theta_{d}|=2$ for PDMS membranes is independent of the method used (Method 1 or Method 2) to monitor the pressure decay and whether *V_ud_* was 11 or 4 cm^4^, provided that the 1st and 2nd corrections were applied to the original data. Therefore, for the first time, we have provided direct validation for determining the time lag from pressure decay data.

## Limitations

The rationale of Method 1 was to record the pressure decay right after the initiation of the experiment. Although the current CV system allows for the reduction of pressure in the reference volume to match the anticipated pressure drop upon initiation of the experiment, the results from the first 5–6 s had to be discarded (as shown in Fig. 4). The initiation of the time-lag experiment is associated with an adiabatic gas expansion in the working volume, which leads to a sudden decrease in gas temperature from *T_i_* to *T_f_*, where:

(7) $$T_{f}=T_{i}{\left(\frac{V_{i}}{V_{f}}\right)}^{\gamma -1}$$

where *V_i_* = *V_w_* is the working volume before starting the experiment, *V_f_* = *V_u_* is the total working volume after starting the experiment, which includes the dead volume, and *γ* is the ratio of the specific heats (*C_p_*/*C_v_*), which for most common gases, including N_2_ is equal to 1.4. As previously discussed, because of the deflection of a metal diaphragm in the differential pressure transducer, the expansion of the working volume was also associated with a slight expansion in the reference volume (by 0.11 cm^3^). Because gas molecules constantly collide with the tube walls, the gas temperature after the initiation of the experiment quickly returns to the initial value (*T_i_*) because the thermal capacity of the gas is negligible compared to the capacity of the tubes containing the gas. This effect is evident in the bottom panel of Fig. 3 (0-5s). Because of the instantaneous cooling and the quick return to *T_i,_* the pressures in the working and reference volumes right after the initiation of the experiment were below the expected values, resulting in unreliable readings by the DPT-1 in the first 5–6 s (Fig. 4). The phenomenon described above is also evident in Method 2 (Fig. 4). The pressures recorded by APT-1 and APT-4 in the working and reference volumes dropped momentarily below the expected values after the initiation of the experiment. However, because the valve connecting the working and reference volumes was closed after 5 s from the initiation of the experiment, i.e., when the gas temperature returned to its initial value, it is believed that the adiabatic expansion did not affect the readings of the DPT-1 in Method 2.

Despite the impossibility of recording a reliable pressure decay in the first 5 s after initiating the experiment in Method 1 and Method 2 for two different reasons, the application of the two corrections discussed in the *Processing of pressure decay data* Section leads to excellent agreement between the upstream and downstream time lags. Method 2 does not require adjusting the pressure in the reference volume to the anticipated gas expansion in the working volume after initiating the experiment; therefore, it is technically more straightforward to use. Because reliable pressure decay results are available after similar times in both methods, Method 2 is recommended over Method 1 for recording the pressure decay in time-lag experiments.

## CRediT authorship contribution statement

**Peter Leszczynski:** Methodology, Validation, Formal analysis, Writing – original draft. **Zheng Cao:** Methodology, Validation, Formal analysis, Investigation, Writing – review & editing. **Haoyu Wu:** Investigation. **Jules Thibault:** Investigation, Writing – review & editing. **Boguslaw Kruczek:** Conceptualization, Funding acquisition, Supervision, Investigation, Writing – review & editing.

## Declaration of competing interest

The authors declare that they have no known competing financial interests or personal relationships that could have appeared to influence the work reported in this paper.

## Data Availability

Data will be made available on request.
